# Another brick in the wall of needs for invasive ventilation?

**DOI:** 10.1186/cc13769

**Published:** 2014-03-17

**Authors:** Michael Quintel, Onnen Moerer

**Affiliations:** 1 Department of Anaesthesiology, Emergency and Intensive Care Medicine, University Medicine, Georg-August-University Göttingen, Robert Koch Strasse 40, 37075 Göttingen, Germany

## Abstract

Ventilator-induced lung injury and ventilator-induced diaphragmatic dysfunction are major complications in mechanically ventilated patients with acute respiratory failure. Invasive ventilation adds a further burden by increasing the risk of infections. An approach that protects both lung and diaphragm is pivotal. Mirabella and colleagues compared conventional controlled ventilation with a mode that combines several potentially lung-protective properties - non-invasively applied neurally adjusted ventilatory assist - in an animal experiment. This approach seemed to be as effective but potentially more lung-protective. Although the experimental setup and results cannot be translated directly to the clinical setting, they should motivate us to further study this innovative approach.

## Introduction

In the previous issue of *Critical Care*, Mirabella and colleagues [1] studied the lung-protective capabilities of non-invasively applied neurally adjusted ventilatory assist (NAVA) in acute respiratory failure (ARF) in an animal study. In ARF, mechanical ventilation is applied to maintain adequate gas exchange, to stabilize the alveolar space, and to unload the respiratory muscles. The price to pay is, first and foremost, ventilator-induced lung injury (VILI). ‘Protective’ strategies using low tidal volumes and limited airway pressures in combination with positive end-expiratory pressure (PEEP) have been able to show a gradual reduction of VILI, thus influencing outcome [2]. In addition to VILI, controlled mechanical ventilation causes atrophic loss of muscle strength because of inactivation and ventilator-induced diaphragmatic dysfunction (VIDD) [3,4]. Both VILI and VIDD have major implications for morbidity and mortality.

NAVA provides airway pressures proportional to the electrical activity of the diaphragm during inspiration, ensures efficient patient-ventilator synchrony, and maintains the degree of biovariability that corresponds to the actual status of the patient [5-9]. These properties not only might be lung-protective but also might enable a more physiologic distribution of ventilation within the lungs, leading to improved matching of ventilation and perfusion.

The (at least) initial use of non-invasive ventilation (NIV) in hypercapnic ARF with respiratory muscle weakness is a well-accepted medical standard; in contrast, its use in hypoxemic failure (adult respiratory distress syndrome, or ARDS) is limited because even a very short loss of end-expiratory pressure might lead to significant derecruitment and severe hypoxemia [10]. Combining the effect of these two different approaches (NIV-NAVA), the study by Mirabella and colleagues provides new conceptual insights into lung-protective ventilation [1].

## Hypothesis, results and open questions

Based on the hypothesis that NIV-NAVA (without external PEEP) might be equally protective, the authors compared it with conventional invasive mechanical ventilation (tidal volume of 6 mL/kg and PEEP of 5 cm H_2_O) with neuromuscular blockade in an animal experiment with acid-induced ARF (arterial partial pressure of oxygen/fraction of inspired oxygen ratio of less than 200) [1]. At 6 hours, both groups recovered to oxygenation values found before injury. Only the NIV-NAVA group demonstrated an improvement in the dynamic compliance; consequently, lower peak and plateau pressures were measured and lung injury was less pronounced in NIV-NAVA as proven by inflammatory reaction and lung histology.

The authors ascribe these effects to the maintenance of spontaneous breathing, upper airway liberation, and variable breathing pattern. The approach of this study is innovative and, to some extent, challenges the iron law that, in ARDS states comparable to those experimentally induced in this study, intubation and controlled mechanical ventilation are the only appropriate options.

The study, though presenting challenging results, has obvious limitations, foremost relating the findings to one well defined, specific intervention. This is true for two reasons. First, the study compares two different modes of mechanical ventilation: volume-controlled mechanical ventilation in paralyzed, deeply sedated subjects and assisted mechanical ventilation applied with NAVA. Second, it compares two different interfaces by which mechanical ventilation is applied: invasive mechanical ventilation via an endotracheal tube and NIV applied via a nasal prong.

A group of non-invasive pressure support ventilated animals would have allowed us to better understand and interpret the results, but (as stated by the authors) this was technically not achievable.

Before open NIV-NAVA can be considered in the clinical setting, a number of questions presented by the complexity of the topic have to be answered:

1. Do the positive effects prevail over a longer period of time? In a setting where positive effects have to persist and side effects might need time to develop their detrimental effects, an observation period of 6 hours is too short.

2. Is persistent tonic activity less injurious than the application of PEEP? PEEP has a direct effect on respiratory muscle load, recruitment, and pulmonary gas exchange; it reduces alveolar cycling and influences the respiratory drive [11]. Tonic activity of the diaphragm describes a persistently increased activity during the expiratory phase [12,13]. The data presented suggest that, in a setting without external PEEP, increased tonic diaphragmatic activity enables recruitment and enlarges end-expiratory lung volume. The observation that increased tonic diaphragmatic activity induced effects comparable to those of controlled mechanical ventilation with a PEEP of 5 cm H_2_O underlines the importance of non-prima vista effects of NAVA that might open new treatment options for respiratory failure. If ARF resolves within a short period of time, preservation of tonic activity might be an effective measure. The long-term effect remains to be elucidated. A prolonged phase of induced tonic activity might fatigue the diaphragm and induce secondary respiratory failure [13]. The increased tonic activity toward the end of the experiment, therefore, should be interpreted with caution. It remains unclear whether open NIV-NAVA outperforms a setting that includes externally applied PEEP. Consequently, Mirabella and colleagues do not suggest the use of zero PEEP in the clinical setting.

3. Are the findings transferable to a more stable severe ARDS and increased respiratory drive? The preservation of spontaneous breathing has marked beneficial effects [14]. However, by increasing the forces applied to the lung, an unsuppressed, severely increased respiratory drive might be harmful [15]. In states of severe hypoxemia, the question of whether to put the patient at rest or to admit his or her own respiratory drive remains unanswered.

Twenty percent regurgitation in the NIV group would be unacceptable in clinical routine. This finding, however, should be interpreted with caution since the sedation level required to enable the animal experiment might account for this side effect.

## Conclusions

This experimental study by Mirabella and colleagues offers interesting insights into the potentials of NIV-NAVA [1]. It is encouraging that NIV-NAVA has effectively maintained a state in which pulmonary gas exchange was seemingly more protective than conventional invasive ventilation. Thus, in a reversible injury with fast recovery and low tendency for derecruitment, open NIV-NAVA seems beneficial since it offers the opportunity to avoid controlled mechanical ventilation and intubation. Being applied with uncontrollable leakage underlines the properties and potentials of NAVA and should encourage further studies with NIV-NAVA to define its indications in the clinical setting.

However, we would like to state that this study challenges accepted limits or standard of care in several ways. In light of the current standard of routine care in patients who resemble the chosen experimental setup (moderate ARDS), the findings should be weighed carefully before being translated to clinical practice. As noted by the authors themselves, NIV should be restrictively used in patients with hypoxemic respiratory failure, and its application without PEEP cannot be advised at this stage.

## Abbreviations

ARDS: Adult respiratory distress syndrome; ARF: Acute respiratory failure; NAVA: Neurally adjusted ventilatory assist; NIV: Non-invasive ventilation; PEEP: Positive end-expiratory pressure; VIDD: Ventilator-induced diaphragmatic dysfunction; VILI: Ventilator-induced lung injury.

## Competing interests

MQ consults for companies that manufacture ventilators, including Dräger Medical (Lübeck, Germany), CareFusion (San Diego, CA, USA), and Maquet Critical Care (Rastatt, Germany), and is remunerated for these consultations. In 2006, OM participated in a research project at Uppsala University, financed by research grants from Göttingen and Uppsala University and supported by Maquet Critical Care.
